# Laparoscopic Surgery: A Systematic Review of Loop and Knot Security, Varying with the Suture and Sequences, Throws, Rotation and Destabilization of Half-Knots or Half-Hitches

**DOI:** 10.3390/jcm12196166

**Published:** 2023-09-24

**Authors:** Philippe R. Koninckx, Anastasia Ussia, Arnaud Wattiez, William Kondo, Armando Romeo

**Affiliations:** 1 Department of OBGYN, Faculty of Medicine, Katholieke University Leuven, 3000 Leuven, Belgium; 2 Department of OBGYN, Faculty of Medicine, University of Oxford, Oxford OX1 2JD, UK; 3 Department of OBGYN, Faculty of Medicine, University Cattolica, del Sacro Cuore, 00168 Rome, Italy; 4 Latifa Hospital, Dubai 9115, United Arab Emirates; arnaud.wattiez@wanadoo.fr; 5 Department of OBGYN, Gemelli Hospitals, Università Cattolica, 00168 Rome, Italy; anastasia.ussia@gmail.com; 6 Department of Obstetrics and Gynaecology, University of Strasbourg, 67081 Strasbourg, France; 7 Centro Avançado de Cirurgia Ginecológica, Curitiba 81020-430, Brazil; williamkondo@gmail.com; 8 Project Leader Research Educational Center, University of Turin, 10124 Torino, Italy; armandoromeo56@gmail.com

**Keywords:** knot sequences, half-knot, half-hitch, knot rotation, knot security, loop security, laparoscopic surgery, gynecology

## Abstract

Surgical knots are sequences of half-knots (H) or half-hitches (S), defined by their number of throws, by an opposite or similar rotation compared with the previous one, and for half-hitches whether they are sliding (s) or blocking (b). Opposite rotation results in (more secure) symmetric (s) knots, similar rotation in asymmetric (a) knots, and changing the active and passive ends has the same effect as changing the rotation. Loop security is the force to keep tissue together after a first half-knot or sliding half-hitches. With polyfilament sutures, H2, H3, SSs, and SSsSsSs have a loop security of 10, 18, 28, and 48 Newton (N), respectively. With monofilament sutures, they are only 7, 16, 18, and 25 N. Since many knots can reorganize, the definition of knot security as the force at which the knot opens or the suture breaks should be replaced by the clinically more relevant percentage of clinically dangerous and insecure knots. Secure knots with polyfilament sutures require a minimum of four or five throws, but the risk of destabilization is high. With monofilament sutures, only two symmetric+4 asymmetric blocking half-hitches are secure. In conclusion, in gynecology and in open and laparoscopic surgery, half-hitch sequences are recommended because they are mandatory for monofilament sutures, adding flexibility for loop security with less risk of destabilization.

## 1. Introduction

Suturing and knot tying are essential skills in surgery. The choice of the suture material and knots was based on common sense until the loop and knot securities were measured with a dynamometer [1].

Understanding loop and knot security is important for the laparoscopic surgeon who cannot use two-handed knot tying for loop security as performed in open surgery. Knowledge of loop and knot stability permits the surgeon to choose the most appropriate suture and knot combination, and secure knots allow using thinner sutures and cutting the tails shorter with less postoperative adhesion formation. More importantly, secure knots will prevent postoperative complications by knots opening inadvertently or not being leakproof after sealing a vessel. Since still poorly documented [2,3,4], we planned to review what is known about loop and knot security in laparoscopic gynecologic suturing.

## 2. Materials and Methods

PubMed was searched for (“1 January 2000” [Date—Publication]: “1 August 2023” [Date—Publication]) AND knot [Title] AND surgery AND English [Language] NOT (orthopaedic OR orthopedic) NOT video NOT Gordian. The titles of the 544 articles found were screened, and the PDFs or, if not available, the abstract of the 53 articles retained were reviewed. Finally, only 37 articles were found to be useful. A subsequent search of “knot security” [Title/Abstract] NOT orthopaedics NOT education NOT learning found 144 articles, and after screening and checking, two more articles were included. Unfortunately, systematic reviews are variably defined [5]. Although all relevant articles since 2000 were reviewed, and knot security is important in surgery, the review was not accepted by Prospero (ID 450731) since systematic reviews of in vitro studies without an outcome of clear relevance to the health of humans are not eligible, according to their definition. Therefore, a Prism flow diagram is not included.

Only suture diameters between 2.0 and 1 are discussed. Only one article reported larger [6] sutures but without changing the message. Institutional review board (IRB) approval was not needed for experiments in vitro not involving humans or animals, as confirmed in writing by the IRB of Leuven University.

## 3. Definitions and the Basics of Knot Tying

### 3.1. Knots Are Sequences of Half-Knots and Half-Hitches

**Figure 1:**
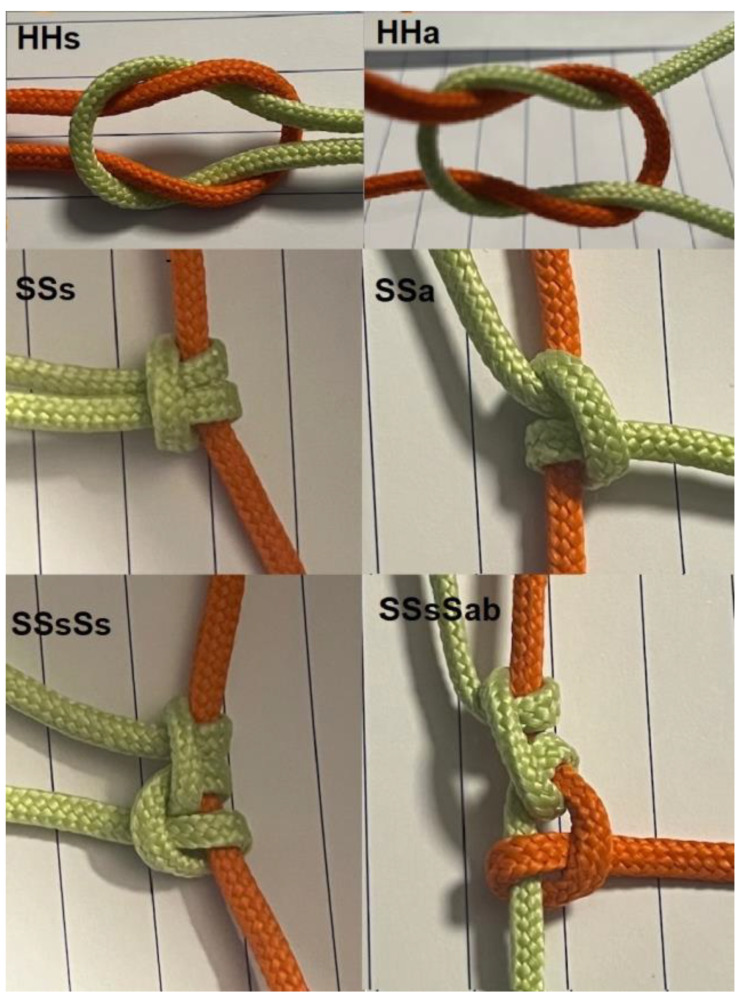
Symmetric and asymmetric Half-knots are easily changed into half-hitches. Three symmetrical half-hitches remain in one plane. When the active and passive ends are changed for a blocking half-hitch, the asymmetric half-hitch becomes symmetric on the new passive end.

In surgery, most knots (for an overview [7]) are sequences of half-hitches (S) or half-knots (H) (Figure 1) Half-hitches result from pulling one end of the suture, called the passive end. Half-knots (H) require symmetrical pulling of both ends. Half-knots are easily converted into half-hitches and vice-versa.

Half-hitches usually consist of one-throw and half-knots of one to three throws.

Besides the number of throws [8], rotation is essential. Compared to the previous one, alternate rotation of the same active end improves clinically knot security [8,9,10,11,12]. Half-knot sequences with alternate rotation result in a flat square knot, visually recognised as symmetrical [9]. Half-hitch sequences with opposite rotation apply a stronger squeeze to the passive end, and both ends stay on the same side. Similar rotation generally results in less secure knots, with the poor granny knot resulting from two one-throw similar rotation half-knots, as an example.

It is important to realize that changing the active and passive ends, as performed in bimanual laparoscopic suturing [13] and blocking half-hitches, is similar to changing the rotation [14].

A different class of knots are cinch knots [15,16], which are complex sliding knots that can be blocked when in place, such as the Röder knot.

### 3.2. Nomenclature of Knots and Historical Perspective

The terminology or nomenclature of half-knots was described in 1976 [17] and of half-hitches in 1984 [12] (Table 1). Half-knots were indicated as “H” and half-hitches as “S” for sliding, followed by the number of throws. For subsequent half-knots or half-hitches, the rotation compared to the previous one was indicated by an “=“ of “x” to indicate a symmetric or asymmetric knot sequence. This terminology felt confusing because “=“ is often called “similar” without specifying that it means an alternate rotation resulting in a symmetric knot and not a similar rotation. For half-hitches, blocking was indicated by // [18].

To avoid ambiguity, we prefer to emphasize rotation because this is what the surgeon does when tying knots [8,9,19]. It is intuitive for the surgeon who has to decide the number of throws and the sense of rotation for each half-knot or half-hitch. Therefore, the H or S is followed by the number of throws and the knot structure, which can be symmetric “**s**” or asymmetric “**a**”, when the rotation of the same active thread compared to the previous one had been alternate or similar. For half-hitches, an “**s**” is added for sliding and a “**b**” for blocking after changing the active and passive ends. It should be clear that a second sliding half-hitch made with a similar rotation, or SSas, can be changed into a blocking half-hitch, or SSab, by pulling the active end. However, it should be equally clear that changing the active and passive ends has the same effect as changing the rotation and that SSab thus indicates a symmetric half-hitch on the new active end. However, for the surgeon, the rules are straightforward: always use alternate rotation for half-knots and sliding half-hitches. Only for blocking half-hitches should similar rotation be used. This terminology can be simplified by omitting the number of throws if one and the “s” if sliding to avoid confusion with the (s) of symmetric. The surgeon also should realise the difference between monomanual and bimanual suturing because similar rotation results in symmetric knots after changing the active and passive ends. Half-hitches remain sliding as long as the passive end remains the same. However, reorganization might block some sequences when six or more half-hitch sequences are used. To summarize, SSsSsbSab indicates a second symmetrical and sliding, a third symmetrical (alternate rotation) and blocking, and a fourth asymmetrical (similar rotation) and blocking half-hitch.

### 3.3. Testing and Definitions of Loop and Knot Security

The security of knots is mostly evaluated with a dynamometer increasing forces at a constant speed and measuring the force when a suture breaks or slides open. Dynamic testing of the security will not be discussed because it is not yet useful in gynecology.

Loop security or holding strength is the first aspect to consider when suturing [20]. This is the force needed to open the first loop, which can be the first half-knot, one or more sliding half-hitches, a sliding granny knot [21], or a cinch knot. Loop security keeps the tissues approximated or sutured arteries leakproof until additional half-knots or half-hitches are added, securing the knot. Loop security, thus, is essential for making tight knots. The importance of inadequate loop security is illustrated by the incidences of postoperative bleeding, which was reported as high as 4% to 10% after hysterectomy in animals [22].

The definition of knot security is variable. Knot security is mostly described as the mean force to slide open the final knot or break the suture. Another definition is the percentage of knots sliding open. A third definition is the percentage of dangerous and insecure knots. Based on clinical experience, these were defined in abdominal and gynecologic surgery as opening with less than 10 N and between 10 and 30 Nexton, respectively. However, the values of insecure and dangerous knots might be much higher for knots on the abdominal fascia or when fixing a mesh to the promontory, especially during coughing. A fourth definition is the percentage of knots sliding for over 3 mm before blocking and breaking the suture. This sliding reflects reorganization when force is applied, but instead of reorganizing into opening the knot, the knot 3-D structure blocks and the suture breaks. This absence of sliding for a few mm is important for sealing vessels so that they are leakproof and in orthopedic surgery requiring tight knots. Although not investigated in gynecologic surgery, the importance today seems to be limited to not cutting sutures shorter than 3 mm. Besides these definitions, knot security after being soaked in water for some time [23] can differ slightly from dry sutures.

## 4. Understanding Knot and Loop Security

### 4.1. Loop Security

Although not measured precisely, loop security can be handled in open surgery by correct two-handed knot tying, with constant tension on both suture ends [24,25]. In laparoscopic surgery, loop security only varies with the friction characteristics of the suture in the tissue and in the knot, reflecting the 3-D geometry in the knot (for mathematical models [24]).

The forces needed in abdominal surgery and gynecology for loop security have not been documented. The only guidance is clinical experience [26], and we (the authors) estimate that the approximation of the abdominal fascia following a laparotomy or the approximation of the myometrium following a myomectomy requires loop security between 30 to 50 Newton (N). Loop security is not an issue for promontofixation because knots are secured without tension. Loop security for pulsatile arteries has not been measured. The need for much higher loop security forces in orthopedic surgery have stimulated the development of cinch knots, or knots sliding on the passive end, that can be blocked by reorganization by traction on the active end. These will not be discussed, being a different class of knots with unclear importance in gynecologic surgery.

Besides the friction forces of the suture in the tissue, the loop security varies with the friction forces of the suture in the knot, varying with the number of throws and the 3D geometry.

**Figure 2:**
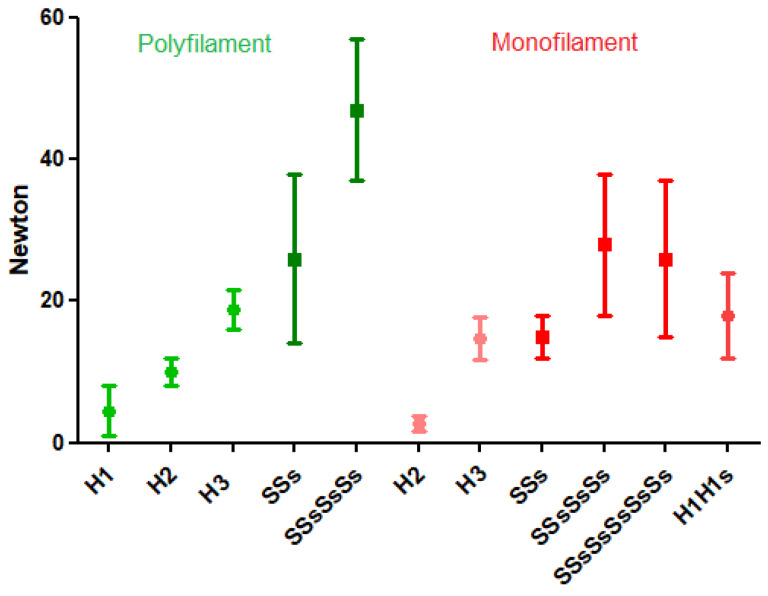
Loop security of polyfilament (Vicryl) and monofilament (Monocryl) sutures. Mean and SDs are indicated. Constructed from [7].

For polyfilament sutures, such as polyglactin 2-0 (Vicryl), the loop security of half-knots increases with the number of throws being 6, 10, and 18 Newton, for an H1, H2, or H3, respectively [8]. The loop security of symmetrical half-hitch sequences is twice as high as asymmetrical sequences [16,27] and increases with the number of half-hitches, being 24 and 48 N for 2 and 4 symmetric (alternate rotation) half-hitches [14]. (Figure 2). Although forces were not measured, the unstable granny knot (H1H1a) and H2H1a can be used to secure veins [28] or for wound closure [21].

For monofilament sutures, such as polyglecaprone 2.0 (Monocryl), the loop security is lower than for polyfilament sutures. The loop security of half-knots was 3 and 15 N for two- and three-throw half-knots. For half-hitch sequences, the loop security was 15 N and 28 N for SSsand SSsSsSs and did not improve when six half-hitches were used (SSsSsSsSsSs) or two half-knots as H1H1s [29].

Figure 2 also shows the variability of the loop security of half-hitch sequences, indicated by the large standard deviations caused by the differences in the forces of tying and duration of time till tested. Only recently, the surgical flexibility of half-hitch sequences was realized. If the loop security of two symmetrical half-hitches (SSs) is insufficient to keep tissues with stronger opening traction approximated as can occur when closing a myomectomy, a third and eventually a fourth half-hitch can be added. In addition, the last or fourth half-hitch can be transformed into a blocking half-hitch by quickly pulling the active end. If the surgeon anticipates this, the fourth half-hitch is preferably asymmetric, made by similar rotation.

The loop security of cinch knots [30,31], although overall much higher than for sliding half-knots or half-hitches, will not be discussed because it is rarely needed in gynecologic surgery. Cinch knots are still under active development to find the best balance between a less complicated knot, high loop security, and how to secure the knot afterwards. Also, the superiority of Millers knot [32,33] or constrictor knot [34] for sealing vessels so that they are leakproof will not be discussed.

### 4.2. Knot Security

**Figure 3:**
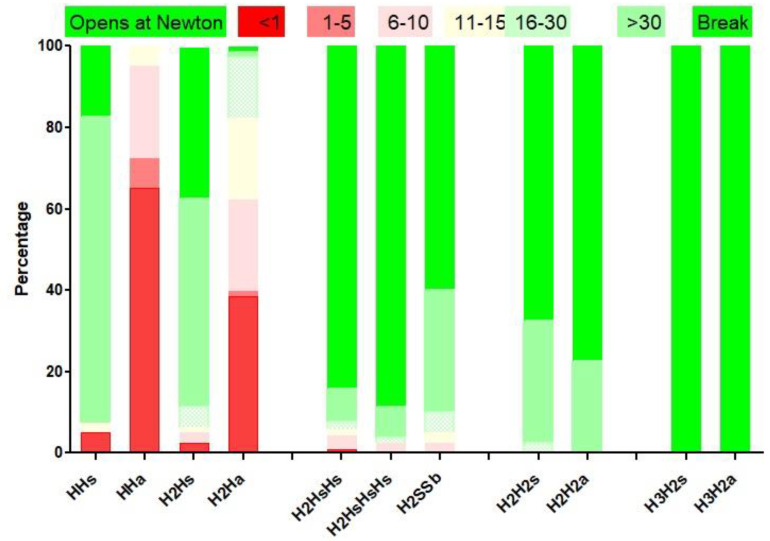
Half-knots security, defined as the percentage of dangerous knots, opening at less than 10 N, and of insecure knots that open between 10 and 30 N. Constructed from [7].

Knot security varies with the type and diameter of the suture, the knot configuration or sequences and rotation of the half-knots or half-hitches, and the number of throws [2,8,14,35]. Knot security is generally slightly lower than the breaking forces of the tread, the knot being the weakest point of a suture [4,8]. This lower breaking force is believed to be caused by angulation forces in the knot (Figure 3). Knot security in vivo can be slightly less because of the wet environment [36] and resorption. 

**Figure 4:**
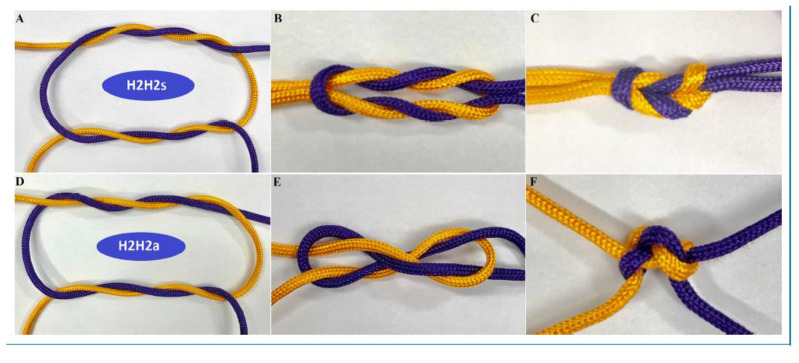
Rotation changes knot reorganization. The symmetric knot remains in one plane when made (**A**), and tying (**B**,**C**). The asymmetric knot is in two perpendicular planes (**D**–**F**). From [7].

Knot security is believed to decrease exponentially over time, halving every 20 days. These considerations are not clinically relevant, considering tissue repair results in 50% of its final strength within a week. Knot security is for most knots occasionally variable because of reorganisation [8,37,38]. Therefore, traditional statistics using the mean and standard deviations are not adequate to evaluate the mean forces breaking or opening the suture. For most knots, some of them slide open due to lower forces and become insecure knots when opening between 10 and 30 N. Occasionally, knots can open at much lower forces below 10 N and are considered dangerous knots. This unpredictable behavior is explained by the reorganisation of knots when forces are applied during tying the knot or when tissue forces try to open the knot. The consequence of reorganization is evident when an H1H1s is transformed into a sliding SSs. The reorganization is also illustrated by the very different reorganization of a double-throw half-knot followed by a symmetric or asymmetric double-throw half-knot (Figure 4). Clinically, reorganization is unpredictable if the first half-knot is slightly destabilized by insufficient loop security, inadvertent traction when making the second half-knot, or asymmetric traction when tying the second half-knot. Unpredictable reorganisation resulted for the excellent surgical knot (H2H1sH1s) in more than 5% insecure knots and 5% dangerous knots [8].

Clinically, the (occasional) risk of insecure or dangerous knots is more important than the mean breaking force of a knot. This variability of knot security is caused by the reorganization of the knot sequences when forces are applied. Equally important is the surgeon who ties the knot. He ties each half-knot or half-hitch with a specific force and maintains this force for a variable time. This introduces the poorly explored memory of the suture or knot as an additional variable. His skills are fundamental for not increasing the risk of accidentally destabilizing a half-knot when making or tying the second half-knot. Also, his training and understanding of knot security are necessary since training and the mental 3-D understanding of a knot structure improve knot tying. An indirect argument is that the relatively high frequency of insecure and dangerous knots, observed in 2018 [8], disappeared progressively with the awareness of the risk of destabilization, the importance of precision in forces used, and the duration of force and suture memory.

Unfortunately, for a risk of a few percent of insecure or dangerous knots, a high number of knots have to be tested. Using a Poisson approximation, 40 and 120 knots will detect 5% abnormality with 95% confidence intervals of 0.6% to 18.0% and 1.8% to 10.9%, respectively [39].

Knot security varies with the number of throws, the rotation and sequences of half-knots or half-hitches, the force of tying [9,40], and the type and diameter of suture used. [4,8].

**Figure 5:**
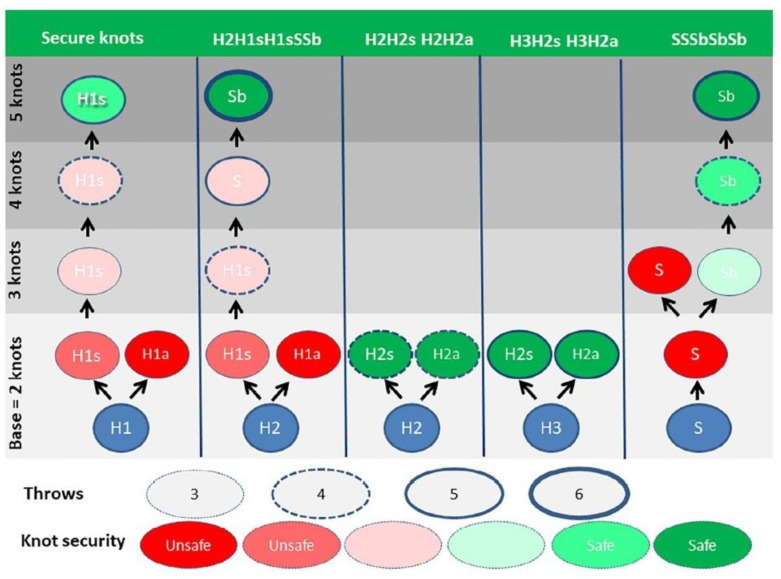
Knot security and number of throws. From [7] with permission.

For polyfilament sutures, secure knots require five throws, whether defined as the percentage opening [4,8,41] or as the percentage insecure and dangerous knots [8]. Knot security, defined as breaking forces, obviously increases with suture diameter. However, security also improves with rotation, with symmetric knots made by alternate rotation being more secure [16]. For example, the asymmetric granny knots (H1H1a) and even the H2H1a [21] are so unstable that they can be considered sliding knots with monofilament sutures. Secure half-knot sequences require four or five throws, and only H2H2 and H3H2 sequences are secure without insecure or dangerous knots. Rotation is clinically unimportant for these complex knots, although asymmetrical sequences have a slightly higher breaking force than symmetrical ones. Surprisingly, the surgical knot (H2H1sH1s) was published as dangerous in 5% and insecure in 15%. However, this was subsequently understood as the risk of destabilizing the first loop by little involuntary traction on one of the ends when making or tying the subsequent half-knots: an H1H1 easily transforms into an S1S1, and an H2H1 into a sliding S2S1, irrespective of rotation. This risk increases with the surgeon’s inexperience when threads are short and knot tying is difficult because of location (Figure 5).

Secure sequences of half-hitches require at least two symmetrical sliding and two or preferably three asymmetrical blocking half-hitches for polyfilament sutures. Monofilament sutures require at least six half-hitches, two symmetrical sliding and four asymmetrical blocking [29].

## 5. Which Knots to Use in Laparoscopic Surgery

### 5.1. Considering Loop Security

**Figure 6:**
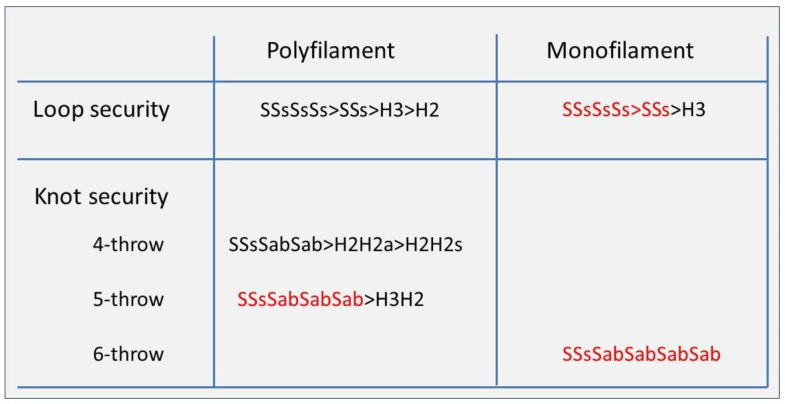
Which knots to use in laparoscopical gynecologic surgery. Secure knots are indicated in black, more secure or preferred knot sequences knots in red.

Estimating that the loop security of more than 15 N is rarely required in laparoscopic gynecologic suturing, an H3, H1H1a, and SSs are acceptable choices for polyfilament sutures. However, with monofilament sutures, the loop security of these sliding knots and of H1H1s (which easily transforms into SSs) might be insufficient. Therefore, we suggest using symmetric half-hitch sequences permitting a third and a fourth half-hitch, eventually blocking if needed. A first double-throw half-knot should be avoided, especially with monofilament sutures (Figure 6).

### 5.2. Considering Knot Security

Secure knots require a minimum of five throws for polyfilament and six throws for monofilament sutures and correct rotation, which is alternate rotation of the same active end or similar rotation if active and passive ends are changed.

Knot reorganization during traction can result in an unpredictable, insecure, or dangerous knot opening with forces of less than 10 N. The risk for this unpredictable behavior increases when the first loop is destabilized because of insufficient loop security or involuntary asymmetric traction on the first half-knot when less experienced or when sutures are short when making or tying the second half-knot. Therefore, as a general rule, we suggest avoiding starting with a double-throw half-knot, especially with monofilament sutures. Replacing half-knot sequences with half-hitch sequences is always advantageous because of higher loop security, more flexibility for loop security if needed, and better or at least equal knot security. This holds for all four-, five- and six-throw knots. For most surgeons, this feels counterintuitive since the surgeon’s knot (H2H1sH1s), and the H2H2 and H3H2, symmetric or asymmetric, are excellent knots.

Secure sequences of half-hitches require at least two symmetrical sliding and two or preferably three asymmetrical blocking half-hitches for polyfilament sutures. Monofilament sutures require at least six half-hitches, two symmetrical sliding and four asymmetrical blocking [29].

With our knowledge of today, knots such as five sequential half-knots only have historical significance by demonstrating that a two-throw half-knot followed by three one-throw half-knots is only slightly superior [4].

### 5.3. Knots and Postoperative Adhesion Formation

Abdominal surgery is frequently associated with postoperative adhesion formation in men and women. Postoperative adhesions are a clinical burden for the patient and society since they cause 30% of postoperative pain, 30% of infertility, and nearly 100% of postoperative bowel obstructions. Peritoneal repair is typically completed within three days, but adhesion formation will occur if repair is delayed by inflammation or a foreign body. Therefore, suture material needing to be resorbed over more than a week is always adhesiogenic. Postoperative adhesions thus increase with the duration of resorption, the suture characteristics, the length of the remaining tails, and the volume of the knot (for review [42]), and thus with the diameter of sutures and the number of throws and half-hitches or half-knots.

Therefore, we wanted to review what is known about loop security because it is important in laparoscopic surgery and knot security. Knot security will permit the surgeon to choose the most appropriate knot and a thin suture with a low knot volume and to cut tails short.

## 6. Discussion

In open surgery, loop security was taken care of with two-handed suturing permitting constant traction of both ends. In laparoscopic surgery, the importance of loop security only became fully realised with the development of laparoscopy in orthopaedic surgery [43], requiring high loop security and tight knots. This has led to the development of a series of sliding cinch knots [44] that can be blocked when in place and subsequently secured with additional half-knots of hitches. Loop security is important for suturing vessels larger than >5 mm to make them leakproof, introducing surgeon’s throw, millers knot, and strangle knots [20], and even this year, an H3 was suggested to initiate an inverted mattress suture in dermatology [45].

In gynecologic laparoscopic surgery, loop security has received little attention because a high loop security is rarely necessary. However, when needed, such as after a large myoma resection, the surgeon should know that monofilament sutures should be avoided and that for polyfilament sutures, an H3 or better two symmetric half-hitches made with alternative rotation should be used. Especially for monofilament sutures, half-hitch sequences have the advantage of being flexible by permitting a third sliding and a fourth sliding half-hitch to be transformed after tying into a securing blocking half-hitch, if needed.

Knot security should be defined by the risk of dangerous or insecure knots, not by the mean breaking forces of the knot. Therefore, the clinical importance of most publications based on mean opening forces of knots can be questioned. Today’s evidence can be summarized as follows: symmetric sequences are overall superior, and knot security increases with the number of throws, requiring four or five for polyfilament sutures and six or more throws of monofilament sutures. With polyfilament sutures, secure half-knot sequences are the four-throw H2H2, the five-throw H3H2 irrespective of rotation, and the surgical knot (H2H1sH1s). However, with monofilament sutures, these half-knot sequences are often insecure, and security does not improve by adding one or more half-knots. Only half-hitch sequences are secure, provided at least four asymmetrical (symmetric on the new passive tread) are used. This indirectly confirms the difference in behavior of monofilament sutures [3]. Considering the risk of reorganization and variability of half-knots, five asymmetrical half-hitches seem a safer option.

This translates into a clear message for the surgeon: always use alternate rotation except for blocking half-hitches. Experienced surgeons can consider H2H2 and H3H2, irrespective of rotation, provided the first loop is not destabilized and has enough security. Half-hitch sequences are superior, especially in gynecology, where they are often sutured deep in the pelvis, because the loop security is far superior to half-knots, and because of the benefit of flexibility, permitting a third and eventually a fourth half-hitch, eventually to be transformed into a blocking half-hitch, when 2 half-hitches do not have sufficient loop security to keep the edges of the tissues approximated.

Variable knot security because of reorganization and destabilization is an insufficiently recognized but serious problem, requiring training, teaching, and individual monitoring. Considering that more than 50 knots need to be evaluated to detect 5% unstable knots, a personal assessment of the knots made by a trainee seems mandatory. As already suggested in 1993 [46], a simple spring portable tensiometer would be welcomed to permit the trainee to check the security of their knots. Another approach is pre-training of laparoscopic psychomotor kills [47], visual force feedback [48], and the use of a knot-tying board with the measurements of the vertical and lateral forces exerted [49]. Also noteworthy is the importance that a trainer cannot be replaced by a video [50], that telementoring and training onsite are equally effective [51], that video registration and artificial intelligence can help in the evaluation of trainees [52], and the importance of the mental image of knots [53] and of fatigue [54]. Ideally, a surgeon should demonstrate minimal skills and knowledge before operating on women, and a structural pre-training [40] and assessing the knot security of 50 to 100 knots with a dynamometer might be a straightforward and reproducible way to evaluate this.

Understanding loop and knot security is also important for training, and expert instruction presents an advantage compared with video-based self-study [50].

Understanding suturing and knot tying is important for loop and knot security and postoperative adhesion formation. Understanding knot security permits using thinner sutures without excessive throws of half-hitches or half-knots out of prudence, and cutting threads shorter results in less adhesion formation. Since 2.0 sutures already have tensile strengths around 80 to 100 Newton, larger diameters are rarely needed in gynecology. Although exact data of forces during coughing are not available, we only can conclude that for securing the mesh to the promontory during promontofixation, sutures with a higher tensile strength might be indicated.

These conclusions are consistent with, but change our understanding of, previous reports. Since the importance of rotation for blocking half-hitches was not clear, it is not surprising that it was concluded that six throws are needed [18,41]. We are also begin to understand reports that multiplying sequences of half-knots do not lead to more secure knots, as demonstrated that four-throw half-knots (H1H1sH1sH1s) [4] with PDS opened in 10%, and suggestions to use five- and six-throw half-knots such as H2H1sH1sH1s and H3H2sH1sH1s [23].

The resorption of sutures is estimated to decrease tensile strength by half over three weeks [55,56]. This is not a concern considering that a tissue repair has 50% of its final resistance after 1 week.

A discussion of modifications and improvements of barbed sutures and of cinch knots, being too complex for intracorporeal suturing [15], is beyond this review.

## 7. Conclusions

In laparoscopic gynaecological surgery, loop security is more important for holding tissues together until the knot is secured than in open surgery, permitting two-handed knot tying with constant traction on the sutures. The loop security of half-knots is low, resulting in many being accidentally transformed into half-hitches with the risk of changing half-knot sequences in sliding half-hitch sequences.

Knots with a very high loop and knot security, as required in orthopedic surgery, are not reviewed despite confirming the importance of three reversing half-hitches [57]. These knots invariably start with a cinch knot reinforced by a series of half-knots or half-hitches, up to double-stranded knot configurations with a loop on one side [58,59] such as the racking hitch knot [60], TSOL knots [61] and Strangler knots.

## Figures and Tables

**Table 1:** Knot sequences are indicated by their geometry. A flat square knot is symmetric and results from an alternate rotation of the same active end. A granny knot is asymmetric and results from a similar rotation of the same active end. Changing the active and passive ends has the same effect as changing the rotation. Knot sequences used to be indicated with = for symmetric (also called identical), X for asymmetric and // for blocking. We prefer a more intuitive indication, with the type of each half-knot (H) or hitch (S), followed by the type of knot, using “s” for symmetric (alternate rotation around the passive end or mono manual suturing) and “a” for asymmetric (similar rotation around the passive tread). For half-hitches, it is indicated whether they are sliding (s) or blocking (b) after changing active and passive ends. For half-hitches, the number of throws is not indicated since it is always one, and sliding is not indicated to avoid confusion with the “s” of symmetric. A two-throw half-hitch results from the transformation of a two-throw half-knot.

Knots	Throws	Knot Sequences	Rotation	Older Indication	New Indication
**Half-knot**	1, 2, 3				H1, H2, H3
		2nd symmetric	alternate	H=H, 1=1	H1H1s, H2H1s, H2H2s, H3H2s
		2nd asymmetric	similar	HxH, 1x1	H1H1a, H2H1a, H2H2a, H3H2a
	**Secure half-knot sequences: H2H1sH1s, H2H2s or H2H2a, H3H2s or H3H2a**				
**Half-hitch**	1, 2				S(1), (S2)
		2nd symmetric	alternate		
		sliding		S=S, 1=1	SSs(s)
		blocking		S//S	SSsb
		2nd asymmetric	similar		
		sliding		SxS, 1x1	SSa(s)
		blocking		S//xS	SSab
	**Secure half-hitch sequences: SSsSabSab**				

## Data Availability

Data ae available from the corresponding author upon request.
